# Contribution of the After-School Period to Children’s Daily Participation in Physical Activity and Sedentary Behaviours

**DOI:** 10.1371/journal.pone.0140132

**Published:** 2015-10-30

**Authors:** Lauren Arundell, Trina Hinkley, Jenny Veitch, Jo Salmon

**Affiliations:** Centre for Physical Activity and Nutrition Research, School of Exercise and Nutrition Sciences, Faculty of Health, Deakin University, Burwood, Australia; Indiana University, UNITED STATES

## Abstract

**Objectives:**

Children’s after-school physical activity (PA) and sedentary behaviours (SB) are not well understood, despite the potential this period holds for intervention. This study aimed to describe children’s after-school physical activity and sedentary behaviours; establish the contribution this makes to daily participation and to achieving physical activity and sedentary behaviours guidelines; and to determine the association between after-school moderate- to vigorous-intensity physical activity (MVPA), screen-based sedentary behaviours and achieving the physical activity and sedentary behaviour guidelines.

**Methods:**

Children (n = 406, mean age 8.1 years, 58% girls) wore an ActiGraph GT3X accelerometer. The percentage of time and minutes spent sedentary (SED), in light- physical activity (LPA) and MVPA between the end-of-school and 6pm (weekdays) was calculated. Parents (n = 318, 40 years, 89% female) proxy-reported their child’s after-school participation in screen-based sedentary behaviours. The contribution that after-school SED, LPA, MVPA, and screen-based sedentary behaviours made to daily levels, and that after-school MVPA and screen-based sedentary behaviours made to achieving the physical activity/sedentary behaviour guidelines was calculated. Regression analysis determined the association between after-school MVPA and screen-based sedentary behaviours and achieving the physical activity/sedentary behaviours guidelines.

**Results:**

Children spent 54% of the after-school period SED, and this accounted for 21% of children’s daily SED levels. Boys spent a greater percentage of time in MVPA than girls (14.9% vs. 13.6%; p<0.05), but this made a smaller contribution to their daily levels (27.6% vs 29.8%; p<0.05). After school, boys and girls respectively performed 18.8 minutes and 16.7 minutes of MVPA, which is 31.4% and 27.8% of the MVPA (p<0.05) required to achieve the physical activity guidelines. Children spent 96 minutes in screen-based sedentary behaviours, contributing to 84% of their daily screen-based sedentary behaviours and 80% of the sedentary behaviour guidelines. After-school MVPA was positively associated with achieving the physical activity guidelines (OR: 1.31, 95%CI 1.18, 1.44, p<0.05), and after-school screen-based sedentary behaviours were negatively associated with achieving the sedentary behaviours guidelines (OR: 0.97, 95%CI: 0.96, 0.97, p<0.05).

**Conclusions:**

The after-school period plays a critical role in the accumulation of children’s physical activity and sedentary behaviours. Small changes to after-school behaviours can have large impacts on children’s daily behaviours levels and likelihood of meeting the recommended levels of physical activity and sedentary behaviour. Therefore interventions should target reducing after-school sedentary behaviours and increasing physical activity.

## Introduction

In spite of the evidence of detrimental health outcomes [1,2], children in developed countries perform suboptimal levels of physical activity (PA) and excessive amounts of sedentary behaviour (SB) [3–5]. Sedentary behaviours has been defined as “any waking activity characterized by an energy expenditure ≤1.5 metabolic equivalents and in a sitting or reclining posture” [6]. Current sedentary behaviours guidelines recommend children in many countries limit their electronic media use/screen-based sedentary behaviours to less than two hours (≤120 minutes) per day [7,8]. In addition, physical activity guidelines state that for health and other benefits children should perform at least 60 minutes of moderate- to vigorous-intensity physical activity (MVPA) every day [7,9,10]. However, in 2011–2012 only 7.8% and 7.1% of Australian 5–17 year old boys and girls, respectively, achieved both of these guidelines (i.e. ≥60 minutes of MVPA/day and ≤120 minutes of recreational screen-based sedentary behaviour/day) [11].

The after-school period, recently defined as the end-of-school to 6pm [12], has been identified as a key period for the accumulation of children’s physical activity and sedentary behaviours [13] and for potential intervention implementation. The varying timeframes previously used to define the after-school period (e.g. 3.30–8.30pm [14] and 4-6pm [15]) and the different locations and contexts of previous research (e.g. attending after-school programs [16]) make direct comparisons between studies of after school behaviours difficult. However, behaviours performed after-school can make a significant contribution to daily activities with up to half of children’s daily steps performed after school [17] and up to 72% of daily TV viewing occurring between 3pm-9pm [18]. Further, evidence shows that the period becomes more important for the accumulation of physical activity as children progress through primary (elementary) and into secondary school [19]. Behaviours performed after school may also contribute to daily physical activity and sedentary behaviour levels, and impact the likelihood of children achieving guidelines for health. Although this is currently unknown, an understanding of how the after-school period contributes to daily physical activity and sedentary behaviour levels and impacts the possibility of achieving guidelines would provide further rationale for interventions to target this period.

Subsequently, the aims of this study were to: 1) identify the percentage of the after-school period boys and girls spend in objectively measured sedentary behaviour (SED), light-intensity physical activity (LPA), MVPA and proxy-reported screen-based sedentary behaviours; 2) identify how the after-school period contributes to daily levels of SED, LPA MVPA and screen-based sedentary behaviour; 3) identify how the after-school period contributes to achieving the physical activity and sedentary behaviours guidelines among boys and girls (i.e. ≥60 minutes of MVPA/day and ≤120 minutes of screen-based sedentary behaviour/day); and 4) examine the association between after-school MVPA and sedentary behaviours and the odds of achieving the physical activity/sedentary behaviours guidelines during weekdays.

## Methods

This study used baseline data from the Transform-Us! intervention (ACTRN 12609000715279; ISRCTN 83725066). Transform-Us! targeted children’s physical activity and sedentary behaviours and the methods for that intervention have been described in detail elsewhere [20]. In brief, 595 grade 3 children (total n = 1606; response rate 37%) were recruited from 20 randomly selected primary (elementary) schools. The schools were within a 50km (31 miles) radius of the city of Melbourne, Australia, had an enrolment of over 300 students, and were stratified by socioeconomic status (SES; 8 of 41 schools in low SES areas and 12 of 96 schools in mid- to high-SES areas). For the current study, the children’s baseline accelerometer data and parent proxy-report data (collected Feb—Jun 2010) were used. Details of each of these measures are provided below.

### Ethics Statement

Ethical approval was obtained from the Deakin University Human Research Ethics Committee, the Victorian Department of Education and Early Childhood Development and the Catholic Education Office. Written parental consent and child assent to complete the assessments were obtained prior to participation.

### Demographics

Children reported their age and sex and parent’s self-reported their highest level of education.

### Accelerometer-assessed physical activity and sedentary time

Children were fitted with a GT3X Actigraph accelerometer (Pensacola, FL) by trained researchers during class time and were informed that the monitors assess how active they are. Accelerometers have been shown to have acceptable validity among children [21]. Children were asked to wear the unit for eight consecutive days (excluding water-based activities and sleep time) on their right hip using an elastic belt. Data were collected in 15-second epochs [22] and downloaded using Actilife Lifestyle Monitoring System, Version 5.1. To determine whether there may be some bias in physical activity levels, children who achieved 3 valid weekdays of accelerometer data were compared with those who achieved 4 or 5+ days; we examined after school and daily MVPA and found no differences in MVPA levels (data not shown).

### Proxy-report after-school screen-based sedentary behaviours

Parents’ proxy reported their child’s behaviour as evidence suggests that children younger than 10 years cannot reliably report the duration of their activities [23]. Parents reported the duration that their child spent during a typical school week (Monday-Friday) after school and during the entire day in the following screen-based sedentary behaviours: 1) watching TV/videos/DVDs; 2) playing Play Station/Nintendo/computer games; and 3) using the computer/internet (excluding games). These survey times were based on reliable survey items from the Children's Leisure Activities Study Survey (CLASS) [23]. The reliability of the modified survey items was assessed through a 7-day test re-test reliability study, with participants from a convenience-sampled Melbourne primary school in 2009 (n = 49, mean age 41.9 ±4.8 years; 75% female). The reliability of the proxy-report screen time behaviours was determined using intraclass correlation coefficients (ICC) which were considered fair (<0.5), moderate (0.5–0.74) or high (≥0.75) [24]. Two of the after-school screen-based sedentary behaviours items showed moderate reliability (ICC: >0.6) and the remaining item (after-school computer/internet) showed fair reliability (ICC: 0.4). Two of the daily screen-based sedentary behaviours items showed fair (ICC >0.4) reliability and the remaining item (playing Play Station/Nintendo/computer games) showed moderate reliability (ICC: >0.6).

### Data management and analysis

Specifically developed excel macros and Stata code (State 12) were used to analyse the raw accelerometer data files. Data from the end-of-school (end-of-school time data collected from the school, mode = 15:30) to 6pm were extracted for analysis. Non-wear time was considered ≥20 consecutive minutes of zero counts in line with the most commonly used definition as identified in a recent review of children’s accelerometer studies [25] and because it provides the most accurate estimate of children’s sitting time [26]. Children were required to have 8+ hours of valid data on at least three weekdays [27] and to have data for at least 50% of the after-school period on each of those days [19] to be included in the analysis of daily behaviours and the after-school period respectively.

Previously established age-specific cut-points [28] were used to calculate children’s LPA (1.6–3.9 METs) and MVPA (≥4 METs) during the after-school period. MVPA was defined as ≥4 METs as calibration studies have shown that the energy expenditure of brisk walking is approximately 4 METs among children and adolescents [29]. SED was defined as <100 counts.min^-1^ which is the cut point that most closely represents sitting time among children [30]. This also corresponds with the Sedentary Behaviour Research Network’s sedentary behaviours definition as sitting has a MET value of up to 1.5 among children [31]. The percentage of the after-school period in which children participated in SED, LPA and MVPA was calculated. The contribution of each of these to daily SED, LPA and MVPA was calculated as: (minutes in intensity during the after-school period/minutes in intensity during the entire day)*100. The contribution that MVPA performed during the after-school period made to achieving the physical activity guidelines on weekdays was calculated as: (minutes in MVPA during the after-school period/60)*100. MVPA data were positively skewed so were log transformed prior to all analyses. To aid in interpretability, the untransformed data were also analysed and results reported.

Mean daily time in after-school screen-based sedentary behaviours was calculated by summing the durations spent in each screen-based sedentary behaviours and dividing by five. The contribution of after-school screen-based sedentary behaviours to total daily screen-based sedentary behaviours was calculated as: (minutes in screen-based sedentary behaviours time during the after-school period/minutes in screen-based sedentary behaviours during the entire day)*100. The contribution that screen-based sedentary behaviours performed during the after-school period made to achieving the sedentary behaviours guidelines (on weekdays was calculated as: (minutes in screen-based sedentary behaviours after-school/120)*100. T-tests were used to assess sex differences between the behaviour intensities. Logistic regression was performed to determine the likelihood of achieving the physical activity guidelines based on the percentage of the after-school period spent in MVPA. Logistic regression was also performed to determine the likelihood of achieving the sedentary behaviour guidelines based on the time spent in screen-based sedentary behaviours during the after-school period. Both models were adjusted for clustering by school and the covariates of age, sex and maternal education. There was no interaction between sex and achieving the physical activity guidelines, therefore all children were analysed together.

## Results

The final sample consisted of 406 children who had valid survey and accelerometer data for the after-school period (68% of sample). Of these, 359 also had valid accelerometer data for the entire day (mean age 8.1 years ±0.47; 58% girls). Three-hundred and eighteen parents (mean age 39.5 years ±5.09; 89% female) completed a survey. There were no differences in age or sex among the children with and without parent proxy-report data. Table 1 shows that both boys and girls spent over half of the after-school period SED (53.7%), almost a third in LPA (32.2%) and 14.1% in MVPA. On average, children spent over an hour SED during the after-school period and approximately 41 minutes in LPA (no sex differences). Boys performed significantly more MVPA after school than girls (18.8 [±9.75] minutes vs 16.7 [±8.03] minutes).

**Table 1:** The mean minutes and percentage of the after-school period spent in sedentary time (SED), light- (LPA) and moderate- to vigorous-intensity physical activity (MVPA), and the contribution to daily behaviour and achieving the physical activity (PA) guidelines.

	All children (n = 406)	Boys (n = 172)	Girls (n = 234)
**Mean percentage of time spent SED and in PA during the after-school period % (±SD)**			
SED	53.7 (±9.96)	54.8 (±10.09)	52.9 (±9.80)
LPA	32.2 (±6.96)	30.4 (±6.67)*	33.5 (±6.86)
MVPA	14.1 (±6.08)	14.9 (±6.26)*	13.6 (±5.89)
**Mean time (mins) spent SED and in PA during the after-school period (±SD)**			
SED	62.3 (±16.82)	62.9 (±16.89)	61.8 (±16.79)
LPA	40.9 (±13.64)	38.6 (±13.25)	42.6 (±13.70)
MVPA	17.6 (±8.85)	18.8 (9.75)*	16.7 (8.03)
**Mean percentage of time spent SED and in PA during the whole day % (±SD)**	**All children (n = 359)**	**Boys (n = 153)**	**Girls (n = 206)**
SED	60.3 (±6.25)	59.9 (±6.33)	60.5 (±6.20)
LPA	29.2 (±4.62)	28.5 (±4.56)*	29.6 (±4.62)
MVPA	10.6 (±3.04)	11.5 (±3.01)*	9.9 (±2.87)
**Mean contribution of SED and PA time during the after-school period to daily behaviour % (±SD)**	**All children (n = 359)**	**Boys (n = 153)**	**Girls (n = 206)**
SED	20.8 (±4.67)	21.4 (±4.97)*	20.4 (±4.38)
LPA	25.0 (±6.08)	24.1 (±6.23)*	25.7 (±5.87)
MVPA	28.8 (±9.35)	27.6 (±9.52)*	29.8 (±9.14)
**Mean contribution of after-school MVPA to achieving PA guidelines** ^**§**^ **% (±SD)**	29.3 (±14.75)	31.4 (±16.25)*	27.8 (±13.38)
Weekday wear time (mean mins/day)	705.6 (±76.7)	717.0 (±78.48)*	697.4 (±74.45)

Sex differences

*p<0.05

^§^physical activity guidelines: ≥60 minutes of MVPA per day.

During the entire day children spent 60.3% of their time SED, 29.2% in LPA and only 10.6% in MVPA. Girls spent a significantly greater percentage of time in LPA than boys, whereas boys spent a greater percentage of time in MVPA than girls during both the after-school period and the entire day. The after-school period accounted for more than one-fifth of children’s daily SED (20.8%) and 27.6% and 29.8% of daily MVPA for boys and girls respectively. The after-school period made a significantly larger contribution to boys’ daily SED than girls’ (21.4% vs 20.4%, p<0.05). Conversely, the period made a significantly larger contribution to girls’ LPA and MVPA than boys’ (LPA: 24.1% vs 25.7%, p<0.05; MVPA: 27.6% vs 29.8%, p<0.05). After school, boys and girls performed 31% and 28% of the MVPA needed to reach the guidelines of ≥60 minutes of MVPA per day.

Significantly more boys than girls achieved the physical activity guidelines (≥60 minutes of MVPA) on weekdays (69.5% vs 47.7% respectively, p<0.01); however, there were no differences in the percentage of boys and girls who achieved the sedentary behaviour guidelines (≤120 minutes screen-based sedentary behaviours) on weekdays (58.7% and 59% respectively). When comparing the percentage of children who achieved both the physical activity and sedentary behaviour guidelines (i.e. ≥60 minutes of MVPA and ≤120 minutes screen-based sedentary behaviours), more boys than girls achieved both guidelines on weekdays (42.7% vs 27.3% respectively, p<0.05).

Data from the parent proxy-report of after-school and daily screen-based sedentary behaviours is shown in Table 2. Boys and girls spent 94.8 (±59.01) and 97.2 (±61.67) minutes, respectively, during the entire day in screen-based sedentary behaviours. After-school boys and girls performed 113.4 (±89.90) and 109.3 (±80.32) minutes respectively of screen-based sedentary behaviours. The after-school period contributed to 84% of children’s daily screen-based sedentary behaviours and 80% of the maximum screen-based sedentary behaviours recommended for children (≤120 minutes/day). There were no sex differences in daily or after-school screen-based sedentary behaviours.

**Table 2:** Parental proxy-reported screen-based sedentary behaviours during the after-school period (mean minutes ±SD), and the contribution to daily screen-based sedentary behaviours (±SD) and achieving the sedentary behaviour (SB) guidelines (±SD).

	All children (n = 318)	Boys (n = 136)	Girls (n = 182)
**After-school screen-based sedentary behaviours** minutes (±SD)	96.16 (±60.45)	94.8 (±59.01)	97.19 (±61.66)
**Daily screen-based sedentary behaviours** minutes (±SD)	111.0 (±84.32)	113.4 (±89.80)	109.3 (±80.32)
**Contribution of after-school screen-based sedentary behaviours to daily screen-based sedentary behaviours** % (±SD)	83.6 (±22.92)	83.2 (±20.93)	83.9 (±24.35)
**Contribution of after-school screen-based sedentary behaviours to achieving the sedentary behaviours guidelines** ^**§**^ % (±SD)	80.1 (±50.38)	78.9 (±49.17)	80.9 (±51.34)

^§^sedentary behaviour guidelines: ≤120 minutes of screen-based sedentary behaviour per day

Results of the logistic regression showed that the odds ratio of achieving the physical activity guidelines was 1.31 (95%CI: 1.18, 1.44, p<0.01). Therefore, for each 1% increase in percentage of the after-school period spent in MVPA, children have a 31% increased likelihood of achieving the physical activity guidelines of ≥60 minutes of MVPA per day. Logistic regression also showed the odds ratio of achieving the sedentary behaviour guidelines was 0.97 (95% CI: 0.96, 0.97, p<0.01). For each 1% increase in screen-based sedentary behaviours during the after-school period, children were 3% less likely to achieve the sedentary behaviour guidelines of ≤120 minutes in screen-based sedentary behaviours per day.

## Discussion

This study highlights the importance of the after-school period for children’s physical activity and sedentary behaviours participation and achieving the associated guidelines. Results showed a 31% increase in likelihood of achieving the physical activity guidelines for every 1% increase in the after-school period spent in MVPA. If this finding was extrapolated for an average child in the current study, a 1% shift (for instance, from 14% to 15% of the after-school period in MVPA) would increase MVPA from 21 minutes to 22.5 minutes during an after-school period of 150 minutes (e.g. 3.30-6pm). We also conducted the analysis with minutes of after-school MVPA as the predictor. Results were very similar and therefore are not presented. After-school screen-based sedentary behaviours participation was associated with a reduced likelihood of achieving the sedentary behaviours guidelines. For every additional 10 minutes of screen-based sedentary behaviours, children are 30% less likely to meet the sedentary behaviours guidelines further highlighting the need to reduce after-school screen-based sedentary behaviours.

Children perform the majority of their daily screen-based sedentary behaviours during the after-school period and almost reach the maximum recommended amount of daily screen-based sedentary behaviours during this period alone. Previous research has investigated if children meet or exceed the sedentary behaviour guidelines on a daily basis [7,11]. However, the current study produces additional data on when the screen-based sedentary behaviours are actually occurring, providing valuable information regarding the time of day that interventions should be implemented.

Although less than 15% of children’s time after school is spent in MVPA, this contributed almost a third of children’s daily MVPA, highlighting the crucial role the after-school period plays in the accumulation of children’s daily physical activity. Although girls spent a lower percentage of the after-school period in MVPA, that percentage made a larger contribution to girls’ total daily physical activity (both LPA and MVPA) than boys’ after-school physical activity. This suggests that the after-school period is a key time of the day to target these behaviours among girls as they perform MVPA at lower levels than boys during other times of the day (e.g. recess and lunchtime [32]). In contrast, although the majority of children’s time was spent sedentary in the after-school period, the contribution of this period to daily sedentary levels was the smallest across the activity intensities measured. Children must therefore also be spending large volumes of time sedentary during other periods of the day, such as school class time [30]. However, there is a great opportunity to reduce sedentary behaviour by targeting the after-school period.

Previous studies have shown boys participate in more physical activity [33,34] and specifically more MVPA after school than girls [35]. This was also found in the current study as boys engaged in MVPA for a greater percentage of the day and the after-school period than their female counterparts. The sex differences was also seen when examining the duration (minutes) of MVPA performed after school. However, girls performed more LPA after school than boys, which may be attributed to active transport or active free play after school. Investigation into the actual behaviours that children perform after school may help us further understand these differences and provide targets for interventions in the after-school period.

In comparison to children in other studies and countries, the current sample spent a smaller percentage of the after-school period in SED, and a greater percentage of time in LPA and MVPA than Canadian 11 year olds [36]. Compared to a sample of children from Sydney (5–7 years), the current sample spent less time sedentary (106 minutes), more time in LPA (90.6 minutes) and more time in MVPA (14.5 minutes) after school [37]; however, the after-school period used among the Sydney sample was longer than the period examined in the current study (3:30-7pm compared to end-of-school to 6pm). The current sample performed similar levels of daily MVPA to 6–19 year old Canadian children (57 minutes/day) [38] but less than New Zealand 10–14 year old children (102 minutes/day) [39]. Further, a greater proportion of the current sample achieved the physical activity guidelines than 7–8 year olds from England (51%) [40], yet fewer achieved them than New Zealand 10–14 year olds [39]. In regards to achieving the sedentary behaviours guidelines, a smaller proportion of the current sample achieved the guidelines than Canadian 5–11 year olds (69%) [41], but a greater proportion achieved them than 11–15 year old Scottish youth (76%) [42]. These variations may be due to sampling and age differences, and could also be due to sociocultural, policy and/or environmental differences between countries. However, purpose-designed representative samples that use a consistent methodology (e.g., the Health Behavior in School-Aged Children survey [43]) are needed to better examine these differences in physical activity and sedentary behaviours estimates during the after-school period between studies (and countries).

This is the first study to determine how important the after-school period is for children’s likelihood of achieving the physical activity and sedentary behaviour guidelines. In particular, findings highlight the large impact that very small changes in after-school physical activity can have on the probability of children meeting the physical activity guidelines (31% more likely to meet the guidelines for every 1% increase in time spent in MVPA during the after-school period). It is therefore important to develop interventions that target increases in children’s physical activity and reductions in sedentary behaviour and screen-based sedentary behaviours during the after-school period. For example, active homework strategies [20] may target both physical activity and sedentary behaviour levels. Introducing after-school dance classes to African-American girls has also been shown to successfully increase physical activity levels and reduce TV/video viewing and video game use [44]; these strategies may be adapted to different target populations. Further, time spent outdoors [45] is consistently shown to be associated with physical activity and the after-school period provides a feasible opportunity to promote outdoor play and subsequently physical activity.

### Strengths and limitations

The response rate was 37% and as such it is not known if these children were representative of the broader sample. As parents are not with their child during some parts of the day (e.g. school hours), they may not be able to recall their screen-based sedentary behaviours during this time which may impact their reporting of daily screen-based sedentary behaviours duration. Further, the objective measure of sedentary time may include time when children are standing and not moving as it is not a direct measure of sitting. Also, the proxy-report measure did not ask specifically for the time engaged in specific sedentary behaviours while sitting. However, the collection of objective measures of physical activity and overall SED and proxy-report screen-based sedentary behaviours duration during the after-school period (when parents are often with their child) is a strength of this study as this provided the intensity and duration of after-school behaviours performed by young children. This information may be used to enhance the effectiveness of interventions which target after-school physical activity and sedentary behaviour. Future research should also examine other contextual factors that may be related to children’s after-school behaviours (e.g. who the child is with, where they are and SES) and collect detailed information on the type of physical activity (e.g. basketball, running) and sedentary behaviours (reading, sitting in a car) they are performing to further tailor after-school interventions.

## Conclusion

After school, children perform 80% of the maximum daily recommendation for screen-based sedentary behaviour but less than 30% of the daily MVPA recommended for health. After-school MVPA is positively associated with achieving the physical activity guidelines and after-school screen-based sedentary behaviours are negatively associated with achieving the sedentary behaviour guidelines. However, more than half of the after-school period is spent in screen-based sedentary behaviours and little time is spent in MVPA. Interventions should target reductions in sedentary behaviour and increases in physical activity during the after-school period as the behaviours performed during this period make a large contribution to daily levels and to achieving the associated guidelines.
